# Enhancing trust in healthcare robotics: implications derived from medical professionals’ perspectives

**DOI:** 10.1007/s11548-026-03755-4

**Published:** 2026-07-28

**Authors:** Nina Müller, Esra Islertas, David Daniel Ebert, Albrecht Schmidt, Dirk Wilhelm

**Affiliations:** 1 Research Group MITI, School of Medicine and Health, TUM University Hospital, Technical University of Munich, Munich, Germany; 2 Munich Institute of Robotics and Machine Intelligence (MIRMI), Technical University of Munich, Munich, Germany; 3 School of Medicine and Health, Graduate Center of Medicine and Health, Technical University of Munich, Munich, Germany; 4 Chair of Psychology and Digital Mental Health Care, Department of Sports and Health Sciences, Technical University of Munich, Munich, Germany; 5 Chair for Human-Centered Ubiquitous Media, Media Informatics Group, Ludwig Maximilian University of Munich, Munich, Germany; 6 Department of Surgery, School of Medicine and Health, TUM University Hospital, Technical University of Munich, Munich, Germany

**Keywords:** Trust, Human–robot interaction, Healthcare, Patient ward, Surgical department

## Abstract

**Purpose:**

Trust is essential for effective teamwork in healthcare, enhancing staff performance, psychological well-being, and the quality of care. With rising workloads and global nursing staff shortages, technologies like robotic systems become increasingly relevant. This contribution examines trust-influencing factors in human–robot interaction in the medical domain.

**Methods:**

To identify implications for our robotic system KIRA-med, we conducted a structured literature search in accordance with PRISMA guidelines. PubMed, IEEE Xplore, and Scopus were searched from January 2015 to December 2025 to identify studies investigating medical staff’s trust in robotic systems. Studies including medical staff like nurses or physicians that reported trust while interacting with a robotic system in the clinical context were included.

**Results:**

Three studies were included, primarily evaluating humanoid or telepresence robots with small cohorts in short-term, controlled laboratory settings. Trust was measured using diverse questionnaires and scales, often as a secondary outcome rather than the primary focus. Identified system requirements to foster trust included introducing the robot to the staff, providing training, and employing expressive cues.

**Conclusion:**

Building trust between medical staff and robotic systems is crucial for their effective use. Trust research on robotic systems such as KIRA-med can help to streamline workflows, reduce staff burden, and free time for direct patient care, enhancing overall care quality.

## Introduction

Trust is a fundamental prerequisite for effective cooperation in social working relationships [14], especially in dynamic and sensitive environments such as hospitals [40]. A study of healthcare professionals showed that trust helps staff solve complex problems, makes teamwork more efficient, promotes motivation and personal and professional growth, reduces psychological stress, and increases satisfaction. Furthermore, trust positively affects patients by increasing the safety and improving the quality of care [12]. To build this trust, employees should, for example, follow the model of *Mayer et al.* [32] and demonstrate ability, benevolence, integrity.

Given these benefits, trust within the healthcare sector is particularly important, since the workload for employees is high and staff are scarce. The WHO predicts a global shortage of up to 4.5 million trained healthcare professionals by 2030 [47]. This problem will intensify in the coming years, both in patient wards and operating rooms (OR) [21]. Stressful working conditions, low wages, and psychological stress, combined with demographic change and an aging society, further worsen the situation [21, 33]. As a result, high-quality patient care cannot always be guaranteed. The number of patients per nurse is increasing, and planned interventions have to be postponed or canceled [5].

In this context, robotic systems are gaining importance. These systems have the potential to relieve medical professionals of some of their daily tasks, giving them more time for direct patient care and thus secure the quality of care. At the same time, building trust between healthcare professionals and such robotic systems remains crucial to ensuring the acceptance and effective use [10] of these technologies. Nevertheless, the perspective of staff and the trust factor in technical developments is largely neglected in research, despite the evidence that clinicians often express low trust in automated systems [16]. To counteract this, our contribution aims to identify research gaps and implications concerning trust, using the KIRA-med assistance robot for patient wards as an example.

## Related work

### Psychological foundations of trust

In psychological trust research, a multitude of concepts to describe trust exist. Due to their prominence and foundational role, the models of Rotter [37] and Mayer [32] are presented, followed by a nursing trust concept [24].

*Rotter* [37] developed a widely recognized concept of interpersonal trust, which is considered the basis of our social order. The definition emphasizes the importance of elements such as commitments between individuals and past experiences [37].

Trust plays an important role not only in private life, but also in organizations, for example, in communication with colleagues. *Mayer et al.* [32] considered trust factors specific for organizations. Ability, benevolence, and integrity are critical elements to build trust, emphasizing the importance of task- or situation-specific skills, action intention, and shared values and norms. Trust, together with perceived risk, influences the risk behavior of the trusting party and, in turn, influences perceived trustworthiness [32].

The continuous cycle model developed by *Johns* [24] focuses on trust within the nursing context. The first step is the assimilation of information relevant to the trust formation. The next step involves “decision-making,” in which perceived trustworthiness and potential positive outcomes play a role. Based on this, a trust relationship is established that is based on various general, process-based, and outcome-based variables. This can lead to varying levels of trust, which in turn influence the assimilation of trust [24]. *Johns’* concept can also be applied to human–robot interaction (HRI) in healthcare. The resulting level of trust is shaped by attitudes toward the system, interaction, and its results, continuously influencing future perceptions and collaboration with the robot.

### Trust in HRI

Despite extensive research on trust in psychology, studies that explicitly measure trust in robotic systems remain limited. This is due to several reasons, one being the lack of a universally accepted definition of trust in the field of HRI. Existing definitions of trust incorporate various factors, such as beliefs and expectations, perceived trustworthiness, prior experience, and the interaction context [29]. Moreover, *Hancock et al.* identify robot-related factors, alongside human and environmental factors, as the most influential and modifiable determinants of trust [22]. Consequently, reliable modeling and measurement of trust in HRI remains challenging [29].

In addition to these challenges, trust in HRI involves both cognitive and affective components. However, many existing studies rely on short, distinct questionnaires without capturing cognitive or behavioral measures associated with trust. Furthermore, numerous measures are developed for individual studies without proper validation [29].

This is demonstrated by the wide range of studies that adapt and apply diverse approaches to measuring trust in HRI. Most focus on technical errors affecting trust, while neglecting social errors, functional robotic systems, and long-term trust assessment [23, 27]. Some studies, such as *Conti et al.* [11] and *Bevilacqua et al.* [6], rely on the *Unified Theory of Acceptance and Use of Technology (UTAUT)* framework to address trust indirectly. Others address trust more explicitly. For example, *Pinto et al.* [35] translate and validate the *Human-Computer Trust (HCT) scale* for a specific language context. While *Bordbar et al.* [7] combine established instruments such as *Negative Attitude torwards Robots Scale (NARS)*, *Affective Learning Scale (ALS)*, and the *HRI Trust Scale (HRITS)* to conduct a multi-component assessment of trust and user attitudes.

Beyond studies that adapt and apply existing measurement instruments, some works also focus on the design of new approaches to measuring trust. Established scales include, for example, the *HCT scale* [30], the *SHAPE Automation Trust Index (SATI)* [19], and the *HRITS* [50]. Nevertheless, such scales come with shortcomings, such as the lack of psychometric tests of construct validity in *SATI* [29]. More recent approaches include *Schaefer* [39], who developed a *Trust Perception Scale for HRI* based on *Hancock’s* [22] three-factor model. During item development, the physical design of a robot was identified as an important yet comparatively underexplored factor in trust formation. The final 40-item scale enables pre- and post-interaction assessment of subjective trust, covering robot capability, behavior, appearance, and task-related aspects, though not explicitly context [38]. *Ullman and Malle* [45] further propose a *Multi-Dimensional Measure of Trust*, which, however, has not yet been validated [31]. The most recent measurement instrument is presented by *Zabel et al.* [51], introducing newly developed measures like the *Attitude Towards Robot Collaboration (ARC) scale*, explicitly capturing cognitive, affective, and behavioral components.

Since trust evolves dynamically throughout HRI, it is crucial to not only measure but also properly model trust [29]. Following the concept of inverse trust, where an autonomous system adapts its behavior to increase the operator’s trust [15], several approaches have been proposed. For instance, *Xu and Dudek* [48, 49] combine objective measures, such as interaction type, with self-reported questionnaire data to dynamically adapt a robots behavior. In addition, more advanced models incorporate multimodal data, including facial expressions, vocal features, and physiological signals such as heart rate, alongside self-reported trust, to capture and model trust more comprehensively [25].

Building on these modeling efforts, trust has been shown to strongly depend on system performance, with reliable systems enhancing operator trust [26, 29]. Furthermore, according to *Gebelli et al.*, robots must be understandable, intuitive, and performant to be usable, allowing healthcare professionals to build trust and rely on them in daily tasks [17].

In this context, trust in robots can be fostered using natural verbal and nonverbal communication to provide clear information to users in a balanced interaction[3, 22, 38]. Nevertheless, linguistic misunderstandings are increasingly common, especially in nursing [27].

To avoid workflow distractions and ensure high-quality care, robotic systems must therefore be carefully implemented and integrated and have reliable, robust, and intelligent communication capabilities [4]. This can be done, for example, according to the principle of human-centered artificial intelligence [13].

### Trust in medical HRI

Robotic assistance systems can be integrated into medical workflows for various purposes, either involved in patient interaction or by performing tasks without direct contact. Regardless of the approach, successful collaboration depends on several key factors, including clear task allocation, communication, and trust [1, 2]. However, trust in robotic systems cannot be assumed, since unfamiliarity and perceived threats may hinder their acceptance within clinical teams [1]. Given that trust in HRI is an inherently multi-dimensional concept including factors, such as transparency, professional, and social characteristics or ethical decision-making [44], its systematic investigation and evaluation between healthcare professionals and robotic systems are crucial for enabling effective collaboration.

Diverse interpretations and assessment approaches to trust known from general HRI are also present in medical contexts. Existing studies often evaluate robots as therapy aids or in long-term patient care scenarios. For example, *Raigoso et al.* [36] assessed the humanoid robot NAO for gait rehabilitation using video-based scenarios, applying the *UTAUT*-based *Almere Model*. Trust was included only as a subscale within a broader acceptance context. Similarly, *Casas et al.* [9] evaluated a social assistive robot for cardiac rehabilitation, where trust was measured through *UTAUT*-based items. Therapists reviewed the system based on a demonstration and discussion.

*Carillo et al.* [8] reported mixed findings when introducing a humanoid robot in pediatric rehabilitation. The evaluation followed the *Acceptance of an assistive social robot questionnaire* that measures trust based on the user’s willingness to follow a robot’s advice. Moreover, *Getson et al.* [18] investigated a non-contact screening robot that for instance monitors a patients temperature. Trust was not directly reported by staff members but rather inferred from their perceived sense of safety.

While these studies demonstrate a growing body of work on robotic systems in healthcare, trust is often addressed only indirectly or as a secondary construct. Furthermore, many studies focus on patient interaction or omit real-world interaction with healthcare professionals and the integration of robotic systems into medical workflows. Therefore, Sections 3 and 4 focus on studies that measure trust between healthcare staff and robotic systems in medical HRI in real-world interactions.

### KIRA-med

KIRA-med (AI-supported robotic healthcare assistance for medical care) is a research project conducted by our research group in which a robotic system is designed to assist with routine tasks, such as taking blood samples or sterile wound dressing changes, in a patient ward. The robot consists of a mobile platform equipped with a robotic arm, whereas the project focuses on natural language interaction between medical staff and the robot, as well as on the collection of contextual information that can be used to dynamically adjust the system’s behavior. The integration of large language models is intended to improve trust-related factors [29], including efficiency, transparency, safety, and acceptance of the system, and to enable seamless integration into the dynamic and sensitive environment.

In this work, KIRA-med serves as an illustrative use case to contextualize the findings of the review. Since trust is a basic prerequisite for successful collaboration, we aim to identify implications for the development, integration, and evaluation of such robotic assistance systems.

## Methods

Our work addresses the challenge of building trust between medical professionals and robotic systems in clinical settings, guided by the following questions:

**RQ1**: *How has trust been studied within HRI in the medical context?*
**RQ2**: *What methods and metrics are used to measure medical staff’s trust in robotic technologies?*
**RQ3**: *What requirements should robotic systems meet to build and foster trust among medical staff?*
**RQ4**: *Which future research directions can be derived from previous studies?*

We assume that research on the trust of healthcare professionals in robotic systems is underrepresented compared to other user groups or work environments. Due to the demands caused by such a dynamic and sensitive environment, we believe that the development and evaluation of trustworthy robotic systems can increase acceptance among medical professionals and thus provide valuable support in the long term.

To answer the questions, we conducted a systematic literature search according to the *PRISMA* scheme [34]. The three electronic databases Scopus, PubMed, and IEEE Xplore were searched for publications in the time period from 2015 to 2025. The search focused on peer-reviewed journal papers and conference articles written in English. We covered combinations of various keywords from the field of healthcare (e.g., healthcare, medical, clinical), robotic interaction and technology (e.g., human–robot interaction/collaboration/communication, healthcare/assistive/medical robot), trust, and target group (e.g., nurse, physician, clinician).

**Fig. 1:**
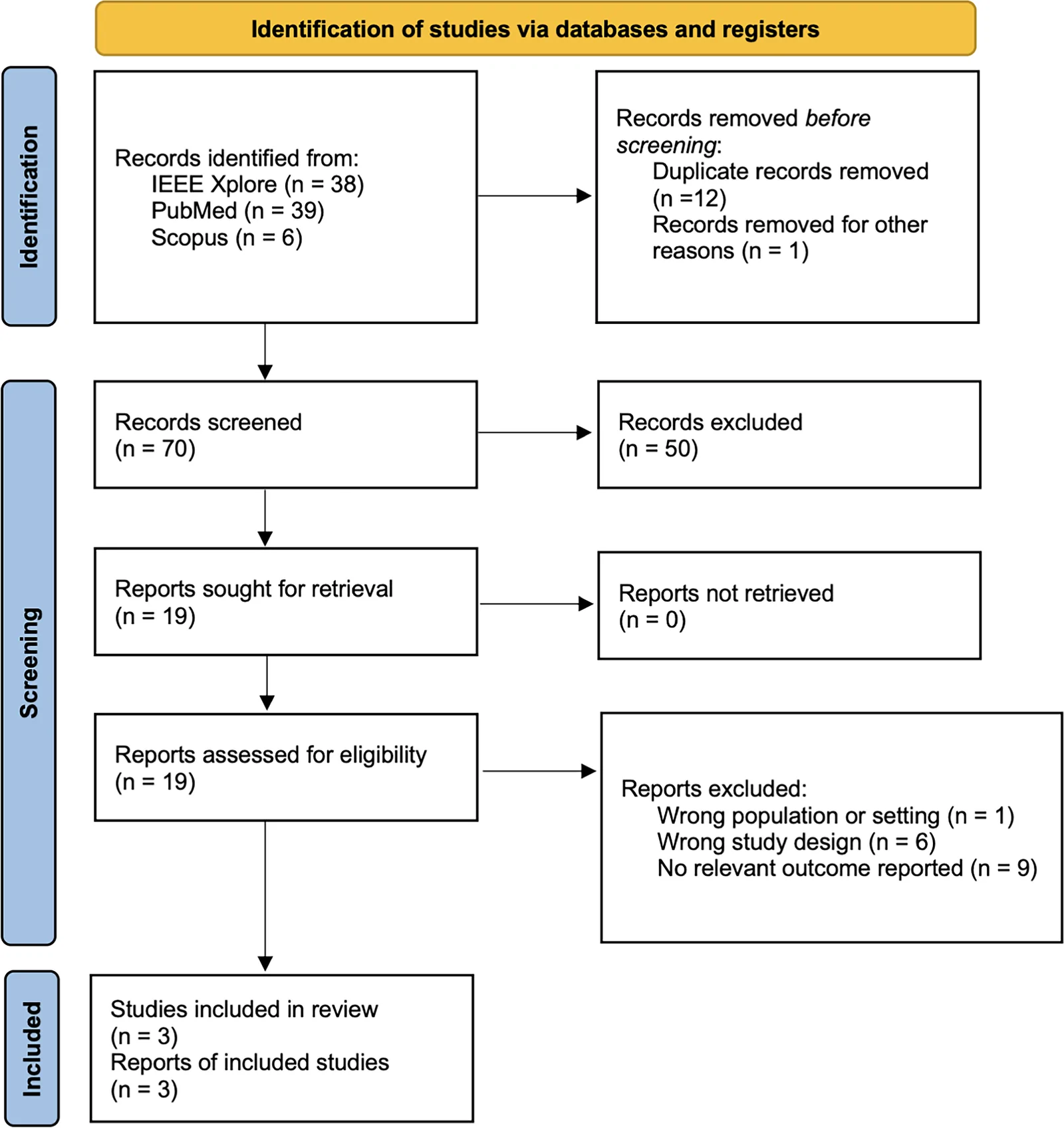
*PRISMA 2020* flow diagram according to [34]

All entries were imported into *Zotero*, and any duplicates found were merged. Two independent reviewers evaluated the remaining entries based on predefined inclusion and exclusion criteria. This process comprised a two-stage screening, consisting of an initial title and abstract screening followed by a full-text evaluation of the retrieved articles. The inclusion criteria required that studies included at least one human participant interacting with a robotic system within the healthcare context. Participants were required to be a medical professional, such as nurses, therapists, physicians, or medical students. Furthermore, the included studies had to evaluated user trust in the robotic system using quantitative, qualitative, or mixed-method approaches, based on user-centered metrics. Review papers and technical feasibility reports without user-centered metrics, studies conducted outside of a healthcare context, studies focusing only on patients rather than medical professionals, and studies not involving an actual robotic system were excluded. Conflicts regarding the inclusion and exclusion criteria were resolved through discussion between the reviewers. For the included studies, the relevant data was extracted, including author, year, study design, number of participants, study environment, robot type, methods used to assess trust, and key findings. The overall selection process is shown in a *PRISMA* flow diagram (see Fig. 1).

## Results

**Fig. 2:**
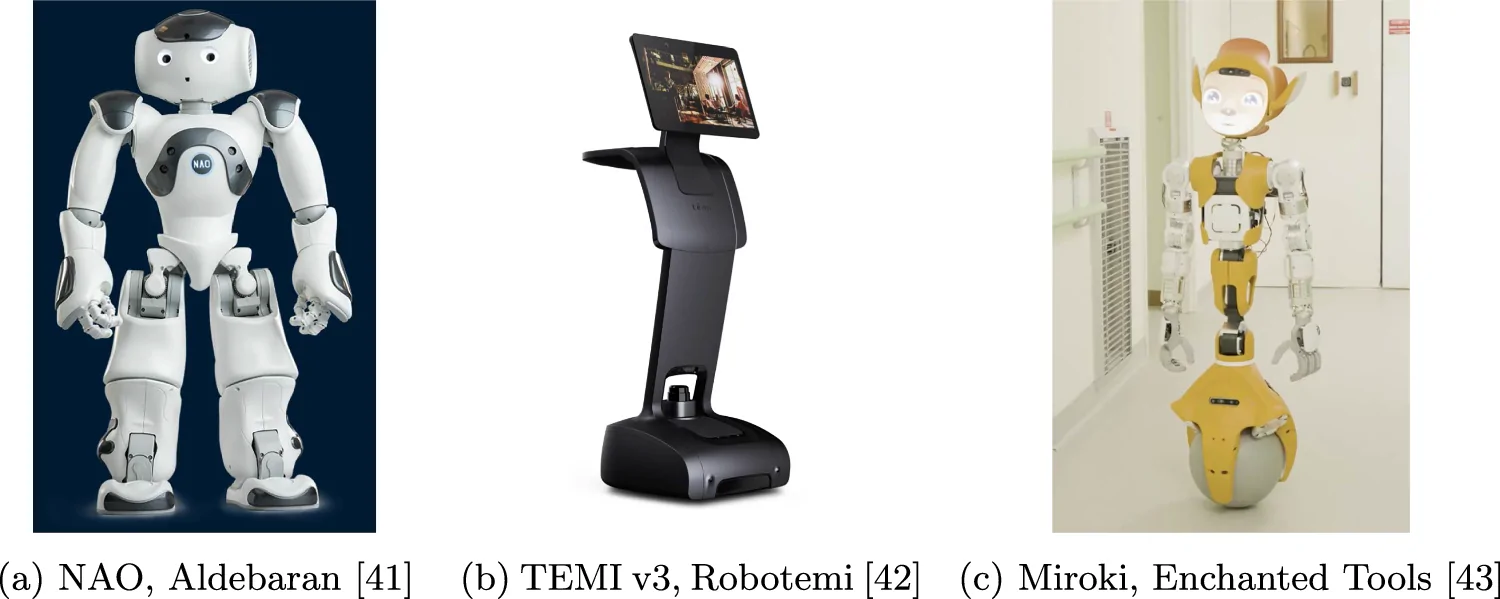
Robotic systems used in the included studies. **a** NAO, Aldebaran [41], **b** TEMI v3, Robotemi [42], **c** Miroki, enchanted tools [43]

Following the PRISMA-based search, three studies were included (see Table 1). The robotic systems used are shown in Fig. 2.

**Table 1:** Summary of identified studies

Author [Ref]	Country	Cohort	Results/conclusion
Gombolay et al. [20]	USA	17 healthcare professionals (physicians and registered nurses)	The acceptance or rejection of robotic compared to computer-based advice for decision-making was observed. Comparisons between computer-based and robotic advice revealed no differences in error rates and similar trust ratings but showed higher attitude ratings for the robotic system
Leoste et al. [28]	Estonia	20 pretest, 5 posttest (healthcare professionals from nursing care clinic)	Results from pretest and posttest questionnaires indicate that trust increases after interactions with the robot in the wild. Recommendations for future research include scenario-based training and iterative development integrating users feedback
Vallée et al. [46]	France	30 healthcare professionals (hospital & nursing care)	Subjective introduction of the robot and high expressiveness of the facial expressions significantly increase trust. The interview trends align with quantitative results. Future research on long-term studies required

*Gombolay et al.* [20] investigated trust and compliance with decision-making advice provided by a computer-based system compared to the humanoid robot NAO. Using Likert-scale questionnaires as a subjective measure, they found no significant differences in compliance between the systems. High-quality advice increased reported trust for both systems, while interaction ratings indicated a preference for the robotic system.

*Leoste et al.* [28] conducted a study with the socially assistive robot TEMI v3 in a nursing care clinic in Estonia. Trust was assessed using pre- and post-interaction Likert-scale questionnaires. In the pretest, 55% of participants rated the robot as trustworthy, 10% as untrustworthy, and 35% were unsure. After two weeks of interaction, 60% rated it as trustworthy and 40% were unsure, with no participants considering the robot untrustworthy [28].

*Vallée et al.* [46] evaluated the effect of introducing the Miroki robot as an object (machine) versus as a subject (Miroki robot) and the degree of expressiveness (low/high) on healthcare staff in a nursing home. Using Wizard-of-Oz control, questionnaires including semantic differentials, and interviews, the researchers assessed whether the robot inspired trust. Results showed that subjective introduction increased trust compared to objective introduction, and high expressiveness further enhanced trust. Interview trends aligned with questionnaire findings but were not statistically significant [46].

Regarding **RQ1**, trust has primarily been studied using humanoid or telepresence robots directly interacting with the medical staff for a short period in a controlled laboratory environment. Only *Leoste et al.* [28] evaluated the robot in a real-world setting over a two-week period. For **RQ2** all studies used their own questionnaires derived from established theories, such as the Trust in Automation Scale or Aubergé’s movement prosody. Responses were collected using semantic differential or Likert-type scales. **RQ3** identified systems requirements including introducing the robot in a subjective manner, providing training sessions for staff, delivering high-quality advice through the robot, and integrating highly expressive facial expressions for humanoid robots to enhance trust. **RQ4** reveals research gaps such as the lack of longitudinal studies with standardized evaluation methods, since existing evidence relies mainly on short-term assessments with diverse measures (see Sect.  Discussion).

## Discussion

Based on the reviewed literature, we synthesize key implications across human-, robot-, and environment-related factors [22] for the development of robotic assistance systems in healthcare the KIRA-med robot. Human-related factors highlight the importance of understanding staff behavior, characteristics, and expectations as underlined by *Abrams et al.* [1], as well as incorporating their preferences during the iterative development process. Tailored training programs appear beneficial to enhance trust [28] by directly addressing engagement and expertise toward the robot and supporting the seamless integration into medical teams. Robot-related factors emphasize the need for increased transparency through end-user-tailored communication and high-quality advice [20], appropriate for the situational context. This may include the use of visual cues or auditory signals. Additionally, further exploration of trust in non-humanoid robot types is needed, as design elements such as facial expressions [46] cannot be implemented while human judgments of the robot capabilities are often influenced by the appearance of the robot [29, 39]. Environmental factors include the establishment of shared mental models based on procedural instructions and context sensitivity. Moreover, long-term evaluation of systems such as KIRA-med in real clinical environments is essential for assessing trust [46].

Building on these findings, several directions for future work emerge. One promising direction is the development of a comprehensive trust framework, grounded in psychological models like *Mayer et al.* [32] or *Hancock et al.* [22] and the insights of this review. This framework can support seamless integration and onboarding of robotic systems in healthcare settings. Moreover, it has the potential to enable context-aware behavior adaptions of robotic systems and therefore facilitate the formation, maintenance, and restoration of trust during long-term HRI. Future work should also aim to establish more unified subjective measures of trust between healthcare personnel and robotic systems in the medical context, such as questionnaires, completed by the integration of objective measures. These approaches could better capture the multi-dimensional nature of trust, as outlined by *Townsend and MajidiRad* [44], while incorporating the specific requirements and contextual characteristics of healthcare environments. Based on findings of *Lewis et al.* [29], we recommend not only assessing trust directly but providing the users with a clear and understandable concept of trust and asking them to describe their own definition. This approach would allow researchers to better contextualize and interpret trust-related questionnaires.

Moreover, future work should investigate how trust in robotic systems influences clinical workflow, staff satisfaction, and patient outcomes. Finally, the transferability of a robotic system that adapts its behavior based on trust assessments to other areas, as the OR, should be explored in further studies.

The limitations of our work include the restriction to three main databases. An expansion to discipline-specific databases was not carried out, as this would have exceeded the intended scope of the contribution. The review focused on a qualitative synthesis to identify implications based on the included studies and does not incorporate a quantitative meta-analysis. Furthermore, the key concept of trust is measured heterogeneously with small cohorts in the included studies mostly as a secondary outcome, thus limiting a direct comparison between the results and generalization. Finally, this study focused on the central theme of trust and therefore did not comprehensively compare all study characteristics with each other.

## Conclusion

We have presented and discussed insights into research on medical staff’s trust in robots in order to draw implications for the development of healthcare robots and identify key issues for future research. In order to enable the seamless integration of robotic systems into clinical practice, it is essential to consider the trust between medical professionals and the system. Trust serves as a prerequisite for safe HRI and collaboration in digital ORs and modern nursing wards. We believe that understanding how and under which circumstances employees trust a system enables the smooth integration and optimization of existing workflows, thereby reducing the burden on employees. At the same time, we are convinced that the quality of care can be improved, as the assistance systems can take over and perform a multitude of different tasks, giving staff more time for direct patient treatment.
